# Screening of Candidate SNPs Potentially Associated with Multi-Parity Litter Size and Litter Weight in Hu Sheep

**DOI:** 10.3390/ani16152365

**Published:** 2026-08-02

**Authors:** Qinghua Zhao, Yuan Gou, Shanhe Wang, Huiguo Yang, Kanati Shalike, Zehu Yuan, Wei Sun

**Affiliations:** 1 Joint International Research Laboratory of Agriculture and Agri-Product Safety of Ministry of Education of China, Yangzhou University, Yangzhou 225009, China; zqh13369328209@163.com (Q.Z.); gou1030923@163.com (Y.G.); shanhe12315@163.com (S.W.); 2 International Joint Research Laboratory in Universities of Jiangsu Province of China for Domestic Animal Germplasm Resources and Genetic Improvement, Yangzhou University, Yangzhou 225009, China; 3 College of Animal Science and Technology, Yangzhou University, Yangzhou 225009, China; 4 College of Veterinary Medicine, Yangzhou University, Yangzhou 225009, China; 5 Institute of Animal Husbandry, Xinjiang Academy of Animal Sciences, Urumqi 830013, China; yanghuiguo3239@163.com (H.Y.); kanati24239@sina.com (K.S.)

**Keywords:** Hu sheep, litter size, litter weight, *BMPR1B*, *TSHR*

## Abstract

Multi-parity records improve ewe evaluation but require long-term follow-up. This study compared litter size, litter weight, and lambing type across four parities in 256 Hu sheep and 90 Tan sheep and evaluated six candidate SNPs in Hu sheep. The SNPs were confirmed by PCR and Sanger sequencing and genotyped using PARMS; repeated records were analyzed with mixed-effects models. The Hu sheep population showed higher litter weight and multiple-birth proportions than the Tan sheep population, together with a higher mean litter size. Analysis of 822 records from 256 Hu ewes showed that both traits increased from the first to the third parity and then remained stable; the same pattern occurred in 156 ewes with complete records. After BH-FDR correction, *BMPR1B* rs418841713 and *TSHR* rs406686139 were significantly associated with both traits. Association directions were unchanged when analyses were restricted to complete records, low-frequency genotypes were recoded, or lambing year was included. In an independent population, combined-genotype differences were nonsignificant, although GGCG had the numerically highest total litter size. An exploratory model indicated that first-parity litter size and both genotypes provided information on subsequent litter weight, but predictive performance was limited. These findings support further validation of *BMPR1B* and *TSHR* in Hu sheep.

## Simple Summary

Improving reproductive performance in sheep requires records that reflect changes across successive parities. In this study, litter size, litter weight, and lambing type were compared across the first four parities in 256 Hu sheep and 90 Tan sheep. Tan sheep were included only as a phenotypic reference, whereas six candidate genetic variants were evaluated in Hu sheep. Analysis of 822 reproductive records from Hu ewes showed that litter size and litter weight increased from the first to the third parity and were similar between the third and fourth parities. The same pattern was observed in 156 ewes with complete records across all four parities. After correction for multiple testing, *BMPR1B* rs418841713 and *TSHR* rs406686139 were associated with both litter size and litter weight. These findings were consistent in complete-case, rare-genotype-recoding, and year-adjusted sensitivity analyses. However, combined-genotype differences were not significant in a separate Hu sheep population, and an exploratory model showed limited predictive performance. These results highlight the value of multi-parity records and support further validation of *BMPR1B* and *TSHR* in larger, independent populations.

## 1. Introduction

Litter size is an important reproductive trait affecting reproductive efficiency and economic returns in sheep, and improving ewe prolificacy is a major objective in sheep genetic improvement. However, litter size is a low-heritability count trait jointly influenced by genetic background, parity, nutritional status, management practices, and environmental conditions. Its low heritability and substantial environmental variation, therefore, limit the accuracy of selection based on a single reproductive record [1,2]. Litter weight reflects both the number of lambs born per litter and their birth weights and is closely related to ewe productivity and the early growth potential of lambs. Accordingly, litter weight should be considered together with litter size to provide a more comprehensive evaluation of ewe reproductive performance [3].

Parity is an important factor affecting ewe litter size and litter weight. As parity increases, ewe age, body condition, reproductive system development, and adaptation to repeated reproduction may change, thereby affecting litter size and litter weight at each parity [1]. However, accurately obtaining consecutive reproductive records across different parities requires long-term tracking of the same group of ewes. Data collection is time-consuming and labor-intensive and is easily affected by culling, missing records, and changes in management conditions. Therefore, existing candidate gene studies on litter size have mostly focused on a single parity, average litter size, or records from only a few parities, while comparisons of consecutive parity changes in litter size and litter weight within the same population remain relatively limited [4,5]. In candidate marker screening, combining multi-parity phenotypic data helps determine whether the effects of candidate loci are stable and also helps evaluate the reference value of early reproductive performance for subsequent production performance.

China has abundant indigenous sheep breed resources, and different breeds show marked differences in reproductive performance. Hu sheep are a typical highly prolific sheep breed in China, characterized by early sexual maturity, year-round estrus, multiple lambing, and suitability for housed feeding [6]. They are commonly used as maternal resources in meat sheep production and crossbreeding [7]. Tan sheep are an important indigenous meat sheep breed in northwestern China, with strong environmental adaptability and good meat quality. However, their reproductive pattern is usually dominated by single lambing, and their litter size is relatively low [8]. Given these contrasting reproductive profiles, the Tan sheep population was included as a phenotypic reference for describing the multi-parity reproductive performance of the Hu sheep population. Candidate-SNP genotyping and association analyses were restricted to Hu sheep. Because the two populations originated from different farms, the observed differences were interpreted as population-level phenotypic differences and could not be attributed exclusively to breed effects.

Advances in genome-wide association studies, whole-genome sequencing, and genomic selection have extended research on sheep reproductive traits from a limited number of major genes to the genome-wide level [9,10]. Nevertheless, allele frequencies and phenotypic effects of reproductive variants may differ among populations because of differences in genetic background, population structure, and linkage disequilibrium. Population-specific evaluation of candidate loci supported by established biological functions or previous association evidence therefore remains valuable for assessing their relevance and consistency across populations and repeated parity records [10]. Using longitudinal reproductive records from Hu sheep, the present study evaluated six candidate SNPs identified through a literature-guided and candidate-region sequencing strategy, rather than attempting to characterize the complete genome-wide architecture of litter size and litter weight.

*BMPR1B*, *BMP15*, and *GDF9* participate in oocyte–granulosa-cell signaling, follicular development, and ovulation regulation and are important candidate genes for prolificacy in sheep [11,12,13]. Previous polymorphism studies in Chinese sheep breeds, including Hu sheep, provided the basis for selecting these genes and their candidate variants for evaluation in the present population [14]. Accordingly, *BMPR1B* rs418841713, *BMP15* rs55628000, and *GDF9* rs160076413 were included in the present study. Among these loci, *BMPR1B* rs418841713 corresponds to the classical FecB fecundity variant and is supported by comparatively strong functional and population-level evidence [11,15]. In contrast, although *BMP15* and *GDF9* have well-established biological roles in sheep reproduction [16], the available evidence is insufficient to establish the specific variants evaluated here as functionally confirmed causal mutations.

*TSHR* is involved in photoperiodic responses, thyroid-hormone signaling, reproductive seasonality, and non-seasonal breeding [17,18]. The missense variant *TSHR* rs406686139 was selected because a previous study combining genome-wide association, selective-sweep, and runs-of-homozygosity analyses identified this locus and reported an association with sheep litter size [18,19]. Nevertheless, its effect in the present Hu sheep population was evaluated as a candidate association rather than assumed to represent a confirmed causal effect.

In addition to these established reproductive genes, *GRID2* was included on the basis of a candidate region identified in a previous genome-wide association study of litter size in East Friesian × Hu crossbred sheep. That study evaluated two *GRID2* variants, g.35685218A>G and g.35685499C>T. These two reported sites were not detected as polymorphic during preliminary pooled-DNA sequencing of the Hu sheep population examined in the present study. Further sequencing of the amplified *GRID2* region identified several intronic SNPs, of which rs410806480 and rs426968202 were selected for subsequent population genotyping [20]. Because direct effects of these intronic variants on *GRID2* expression, splicing, or reproductive function have not been demonstrated, they were treated as exploratory candidate markers rather than confirmed functional or causal variants. Except for FecB, potential associations involving the remaining loci may reflect either direct effects or linkage disequilibrium with nearby functional variants and therefore require independent replication and further functional validation.

Based on this, Hu sheep and Tan sheep were used as the study animals in the present study. First, litter size, litter weight, and lambing type from the first to the fourth parity were compared between the two breeds, and changes in litter size and litter weight across consecutive parities were further analyzed in Hu sheep. Subsequently, six candidate SNPs, including *BMPR1B* rs418841713, *BMP15* rs55628000, *GDF9* rs160076413, *GRID2* rs410806480, *GRID2* rs426968202, and *TSHR* rs406686139, were amplified, validated by sequencing, and genotyped. Their genetic diversity and associations with litter size and litter weight in Hu sheep were analyzed using repeated-measures models. Finally, the combined genotypes of *BMPR1B* and *TSHR* were preliminarily evaluated in an independent population, and the explanatory and internal predictive value of first-parity litter size and candidate genotypes for subsequent litter weight performance was explored. This study aimed to screen candidate SNPs associated with litter size and litter weight in Hu sheep and to provide a reference for subsequent validation of candidate reproductive markers and combined-genotype patterns in Hu sheep.

## 2. Materials and Methods

### 2.1. Ethical Statement

The animal experiments in the current study were reviewed and approved by the Experimental Animal Ethics Committee of Yangzhou University (approval number 202103279, approval date 8 March 2021).

### 2.2. Sample Collection

This study was conducted from 2020 to 2024 at the Suzhou Breeding Farm and Ningxia Breeding Farm. All experimental animals were healthy adult ewes. Ewes at both farms were maintained under housed conditions, naturally mated, and bred throughout the year. Detailed records on diet composition, individual feed intake, body condition, and farm-specific health-management procedures were not available for the present analysis. A total of 256 Hu ewes were obtained from the Suzhou Breeding Farm, and 90 Tan ewes were obtained from the Ningxia Breeding Farm. Reproductive records from the first to the fourth parity were collected for both populations. For Hu sheep, eligible litter-size and litter-weight records were available for 256, 222, 188, and 156 ewes at the first, second, third, and fourth parities, respectively, resulting in a total of 822 parity-level records. Among these animals, 156 Hu ewes had complete records across all four parities. All 822 available records were included in the primary longitudinal analyses, whereas the 156 ewes with complete four-parity records were used for complete-case sensitivity analyses.

A separate Hu sheep population comprising 188 ewes was used for the preliminary combined-genotype analysis of *BMPR1B* rs418841713 and *TSHR* rs406686139. Phenotypic records and individual-level genotypes in this population were obtained using the same trait definitions and PARMS genotyping procedures as those used for the primary Hu sheep population. First-parity litter-size records were available for all 188 ewes, whereas 88 and 24 ewes had records at the second and third parities, respectively. No fourth-parity records were included in this analysis. The formal combined-genotype comparison was restricted to the three mutually exclusive groups GGCC, GGCG, and GGGG. The *BMPR1B*-GG and *TSHR*-CG groups were summarized only as descriptive single-locus references and were not included in the same statistical comparison. Tan sheep were included as a phenotypic reference population for comparisons of litter size, litter weight, and lambing type, whereas candidate-SNP genotyping and association analyses were restricted to Hu sheep. Because the Hu and Tan sheep populations originated from different farms, breed and farm effects could not be separated. Therefore, the observed differences were interpreted as population-level phenotypic differences rather than as effects attributable exclusively to breed. The 256 Hu ewes included in the primary candidate-SNP association analyses were derived from the same breeding flock.

Approximately 5 mL of whole blood was collected from the jugular vein of each Hu ewe into vacuum blood-collection tubes containing EDTA as an anticoagulant. The samples were labeled and stored at −80 °C until genomic DNA extraction.

### 2.3. Phenotypic Data Collection and Processing

Reproductive records from the first to the fourth parity were collected and processed for Hu sheep and Tan sheep ewes, including litter size, litter weight, and lambing type. Litter size was defined as the total number of lambs born in each lambing event. Litter weight was defined as the summed birth weight of all lambs born in the same lambing event. Average lamb birth weight (ALBW) was calculated by dividing litter weight by litter size for the corresponding lambing event. Lambing type was classified as either a singleton or multiple birth, with a litter size of 1 classified as a singleton birth and a litter size of ≥2 classified as a multiple birth. Records were considered eligible for analysis when the ewe identity, parity, litter size, and corresponding litter weight could be matched unambiguously. Records with missing essential information or values that could not be verified against the available farm records were not included in the analysis. Repeated observations from the same ewe were linked using the individual animal identifier. Missing records at later parities were treated as missing and were neither imputed nor coded as zero. The subset of 156 Hu ewes with complete litter-size and litter-weight records across all four parities was used for analyses requiring balanced four-parity records and for complete-case sensitivity analyses.

### 2.4. DNA Extraction

Peripheral blood leukocytes were isolated from EDTA-anticoagulated whole-blood samples, and genomic DNA was extracted using the phenol–chloroform method [21]. DNA purity and concentration were measured using a NanoReady microvolume UV–Vis spectrophotometer (Hangzhou LifeReal Biotechnology Co., Ltd., Hangzhou, China). DNA samples with an A260/A280 ratio of 1.8–2.0 and a concentration greater than 20 ng/μL were used for subsequent molecular analyses. DNA samples were stored at −80 °C until use.

### 2.5. Primer Design and PCR Amplification

Candidate gene and locus selection followed a literature-guided strategy. *BMPR1B* rs418841713, *BMP15* rs55628000, and *GDF9* rs160076413 were selected on the basis of previous studies of fecundity-gene polymorphisms in Chinese sheep breeds [8]. *TSHR* rs406686139 was selected based on previous genomic and association evidence for sheep litter size and non-seasonal breeding [19]. *GRID2* was included on the basis of a candidate region identified in a previous genome-wide association study of litter size in East Friesian × Hu crossbred sheep [20]. The two *GRID2* loci genotyped in the present study, rs410806480 and rs426968202, were selected after sequencing of the amplified *GRID2* region because the two previously reported positions were monomorphic in the preliminary screening of the present Hu sheep population. The detailed biological rationale and prior evidence for these genes and loci are provided in Section 1.

The PCR primers used in the present study were designed de novo and were not adopted directly from the cited studies. Flanking sequences of the target regions were retrieved from the National Center for Biotechnology Information database based on the *Ovis aries* reference genome assembly ARS-UI_Ramb_v3.0 (RefSeq accession: GCF_016772045.2). PCR primers were designed using Primer Premier 5.0 software (PREMIER Biosoft International, Palo Alto, CA, USA) and synthesized by Tsingke Biotechnology Co., Ltd., (Nanjing, China).

The 256 Hu sheep genomic DNA samples were combined into five DNA pools, with approximately 50 individual DNA samples included in each pool. The pooled genomic DNA samples were used as templates for PCR amplification of the candidate gene fragments. PCR amplification was performed in a final volume of 50 μL containing 25 μL of 2× TSINGKE Master Mix (Tsingke Biotechnology Co., Ltd., Beijing, China), 1 μL each of the forward and reverse primers at 10 μM, 2 μL of pooled genomic DNA, and 21 μL of ddH_2_O. The final concentration of each primer was 0.2 μM. The thermal cycling program consisted of initial denaturation at 94 °C for 3 min, followed by 34 cycles of denaturation at 94 °C for 30 s, annealing at the locus-specific temperatures listed in Table 1 for 30 s, and extension at 72 °C for 45 s, with a final extension at 72 °C for 5 min. The PCR products were separated by electrophoresis on 2% agarose gels stained with Green Fluorescent Nucleic Acid Stain (Solarbio Science & Technology Co., Ltd., Beijing, China) and visualized using a ChemiDoc™ Touch Imaging System (Bio-Rad Laboratories, Inc., Hercules, CA, USA). PCR products showing a single predominant band of the expected size were selected for subsequent Sanger sequencing. Primer sequences, annealing temperatures, and expected amplicon sizes are presented in Table 1.

### 2.6. SNP Identification and Genotyping

The 256 Hu sheep genomic DNA samples were combined into five DNA pools, with approximately 50 individual DNA samples included in each pool. PCR products amplified from the five pooled DNA templates were subjected to Sanger sequencing. The pooled-DNA sequencing step was used only for preliminary screening of the candidate regions and was not used to determine individual genotypes or estimate genotype and allele frequencies. The sequencing results verified the target nucleotide positions of the four preselected loci in *BMPR1B*, *BMP15*, *GDF9*, and *TSHR*. The two previously reported *GRID2* positions, g.35685218A>G and g.35685499C>T, showed no detectable polymorphic signal in the pooled sequencing chromatograms. Further examination of the amplified *GRID2* regions identified several intronic variants, of which rs410806480 and rs426968202 were selected for subsequent individual-level PARMS genotyping. Because pooled sequencing may fail to detect very-low-frequency variants and cannot resolve individual heterozygosity, all formal population-genetic and association analyses were based on individual-level PARMS genotyping.

Genotyping of the candidate SNP loci was performed by Wuhan Jingtai Biotechnology Co., Ltd. (Wuhan, China) using the PARMS method. This method is based on allele-specific amplification and fluorescence signal detection. Genotypes at the target loci were determined using SNP allele-specific primers, universal fluorescent primers, and a common reverse primer. Individual genomic DNA samples from the 256 Hu ewes were used separately as templates for PARMS genotyping. The same individual-level PARMS procedure was used to determine *BMPR1B* rs418841713 and *TSHR* rs406686139 genotypes in the separate Hu sheep population used for the preliminary combined-genotype analysis. Genotyping results were interpreted based on fluorescence signal cluster plots and were used for subsequent genetic diversity analysis, association analysis, and combined genotype analysis. Samples without reliable genotype calls were recorded as missing. Information on the SNP genotyping primers is shown in Table 2.

### 2.7. Statistical Analysis

All statistical analyses and graphing were performed using R software version 4.6.1. The glmmTMB (version 1.1.14), nlme (version 3.1-169), and emmeans (version 2.0.4) packages were used for mixed-model analyses and post hoc comparisons, and ggplot2 (version 4.0.3) was used for data visualization. The principal R functions, model specifications, and multiple-testing procedures used in the final analyses are provided in the Supplementary Methods. The SNP genotyping results were processed to calculate genotype frequency, allele frequency, expected heterozygosity (He), observed heterozygosity (Ho), effective number of alleles (Ne), and polymorphism information content (PIC) for each candidate SNP locus. Genotype frequencies were calculated using the number of successfully genotyped individuals at each locus as the denominator. The number of missing genotype calls was also recorded. Deviations from Hardy–Weinberg equilibrium were evaluated using an exact test. A *p* value > 0.05 was interpreted as no significant evidence of deviation from Hardy–Weinberg equilibrium. Based on PIC values, loci were classified as showing low polymorphism (PIC < 0.25), moderate polymorphism (0.25 ≤ PIC < 0.50), or high polymorphism (PIC ≥ 0.50).

(1) $$PIC=1-\sum_{i=1}^{n}\;P_{i}^{2}-\sum_{i=1}^{n-1}\;\sum_{j=i+1}^{n}\;2P_{i}^{2}P_{j}^{2}$$

(2) $$He=1-\sum_{i=1}^{n}\;P_{i}^{2}$$

(3) $$Ne=\frac{1}{\sum_{i=1}^{n}\;P_{i}^{2}}$$

Continuous traits were summarized as the mean ± standard deviation, whereas lambing type was summarized as counts and percentages. Litter size was summarized descriptively by population and parity because no variation in litter size was observed within the Tan sheep population. Between-population differences in lambing pattern were formally evaluated using the singleton-versus-multiple-birth comparison described below. Litter weight was compared between the Hu and Tan sheep populations using a heteroscedastic linear mixed-effects model. Population, parity, and their interaction were included as fixed effects, and Animal_ID was included as a random intercept. Population differences were estimated separately within each parity, and the four parity-specific *p* values were adjusted using the Holm method. Because no multiple births were recorded in the Tan sheep population, the distributions of singleton and multiple births were compared between the two populations within each parity using Fisher’s exact test. The four parity-specific *p* values were adjusted using the Holm method.

Parity-related changes in litter size and litter weight in Hu sheep were analyzed using all 822 available records from 256 ewes. Litter size was analyzed using a zero-truncated Conway–Maxwell–Poisson generalized linear mixed model, with parity included as a fixed effect and Animal_ID as a random intercept [22]. Litter weight was analyzed using a linear mixed-effects model, with parity included as a fixed effect and Animal_ID as a random intercept. A varIdent variance structure was incorporated to allow residual variances to differ among parities [23]. For the primary analyses, the overall effect of parity was assessed using likelihood-ratio tests comparing models with and without the parity effect. For litter weight, models used for likelihood-ratio testing were fitted using maximum likelihood, whereas the final model used for estimation was fitted using restricted maximum likelihood. Estimated marginal means and their 95% confidence intervals were calculated, and pairwise comparisons among parities were adjusted using the Tukey method. The same model structures were fitted to the subset of 156 ewes with complete records across all four parities. In the complete-case analysis, the overall parity effect for litter size was assessed using a likelihood-ratio test, whereas the overall parity effect for litter weight was evaluated using a marginal Wald F test from the final restricted-maximum-likelihood model. Model-selection results, diagnostic assessments, and sensitivity-analysis results for the litter-size and litter-weight models are provided in Supplementary Table S2.

Spearman rank correlation analysis was used to evaluate the relationships among litter size, litter weight, and average lamb birth weight across the first four parities in Hu sheep. The 12 trait-by-parity variables generated 66 unique pairwise correlation tests. Correlations were calculated using pairwise complete observations, and the resulting 66 *p* values were jointly adjusted using the Benjamini–Hochberg false discovery rate (FDR) procedure.

Repeatability of litter weight was estimated from the between-ewe variance and the parity-specific residual variances obtained from the heteroscedastic linear mixed-effects model. For parity k, repeatability was calculated as:

(4) $$R_{k}=\frac{\sigma_{ewe}^{2}}{\sigma_{ewe}^{2}+\sigma_{e,k}^{2}}$$

where $\sigma_{ewe}^{2}$ represents the between-ewe variance and $\sigma_{e,k}^{2}$ represents the residual variance at parity k. Litter-size repeatability was not reported because no validated variance-partitioning definition was available for the final zero-truncated Conway–Maxwell–Poisson model.

Associations between candidate SNPs and repeated litter-size or litter-weight records in Hu sheep were evaluated using mixed-effects models. For each SNP, litter size was analyzed using a zero-truncated Conway–Maxwell–Poisson generalized linear mixed model, whereas litter weight was analyzed using a heteroscedastic linear mixed-effects model with parity-specific residual variances. Parity, genotype, and the parity-by-genotype interaction were initially included as fixed effects, and Animal_ID was included as a random intercept to account for repeated observations from the same ewe.

For each SNP and trait, the model containing the parity-by-genotype interaction was compared with the corresponding model without the interaction term using a likelihood-ratio test. When the interaction was not significant after multiple-testing correction, the model without the interaction term was used for subsequent inference. The overall genotype effect was then tested by comparing the model containing parity and genotype with a reduced model containing parity alone. For litter-weight analyses, models used for likelihood-ratio testing were fitted using maximum likelihood, whereas the selected final models were refitted using restricted maximum likelihood to obtain effect estimates, estimated marginal means, and 95% confidence intervals.

Extremely rare genotypes were not retained as separate groups in the primary inferential models. For *BMPR1B* rs418841713, only the AG and GG genotypes were compared because only one ewe had the AA genotype. For *BMP15* rs55628000, the CT and TT genotypes were compared because only three ewes had the CC genotype. For *GRID2* rs410806480, the AG and GG genotypes were compared because only one ewe had the AA genotype. All three observed genotypes were retained for *GDF9* rs160076413 and *TSHR* rs406686139. *GRID2* rs426968202 was nearly fixed in the study population and was therefore summarized descriptively but excluded from formal association testing.

The genotype main-effect *p*-values from the five formally tested SNPs across litter size and litter weight constituted a family of 10 tests and were adjusted using the Benjamini–Hochberg false discovery rate procedure. The corresponding 10 parity-by-genotype interaction tests were adjusted as a separate family. Estimated marginal means and their 95% confidence intervals were obtained using the emmeans package (version 2.0.4), and genotype pairwise comparisons were adjusted using the Tukey method.

To assess the robustness of the primary SNP-association findings, three complementary sensitivity analyses were conducted: restriction of the analysis to ewes with complete records across all four parities, alternative genotype coding in which extremely rare homozygous genotypes were merged with the corresponding heterozygous group, and additional adjustment for lambing year. Detailed comparisons are provided in Supplementary Table S7.

Parity was included as a fixed effect in all longitudinal models. Lambing year was added as a categorical fixed effect only in the year-adjusted sensitivity analysis. All genotyped Hu ewes originated from the same breeding flock; therefore, farm did not vary in the within-population SNP-association analyses.

For overall model tests that were not included in a multiple-testing family, *p* < 0.05 was considered statistically significant. When multiple comparisons or multiple hypothesis tests were performed, significance was determined using adjusted *p*-values. Tukey-adjusted *p*-values were used for pairwise comparisons among parities and genotype groups. Holm-adjusted *p*-values were used for the four parity-specific comparisons of litter weight and lambing type between the Hu and Tan sheep populations. Benjamini–Hochberg FDR-adjusted *p*-values were used for the 66 correlation tests and the candidate-SNP association tests. An adjusted *p*-value < 0.05 was considered statistically significant.

In the independent Hu sheep population, litter-size traits were compared among the three mutually exclusive *BMPR1B*–*TSHR* combined-genotype groups (GGCC, GGCG, and GGGG) using the Kruskal–Wallis test. The *BMPR1B*-GG and *TSHR*-CG groups were presented only as descriptive single-locus references and were not included in the same statistical comparison. A *p*-value < 0.05 was considered statistically significant, and “ns” indicated *p* ≥ 0.05.

To explore the explanatory contribution of early reproductive records and candidate genotypes to variation in subsequent litter weight performance in Hu sheep, total litter weight from the second to the fourth parity, calculated as the sum of litter weights at parities 2–4, was used as the dependent variable, and a fixed-effect linear model was fitted. First-parity litter size, *BMPR1B* rs418841713 genotype, and *TSHR* rs406686139 genotype were included in the model as categorical fixed factors. Only the main effects of these three factors were included in the model. First-parity litter size of one lamb, the *BMPR1B* AG genotype, and the *TSHR* GG genotype were specified as the reference levels. Because the number of individuals with the *BMPR1B* AA genotype was extremely small (*n* = 1) and some individuals had missing *TSHR* genotypes, individuals with the *BMPR1B* AA genotype and those with missing *TSHR* genotypes were excluded from the analysis. Finally, 154 Hu sheep with complete records across all four parities and valid genotypes were included. The fixed-effect model was as follows:

(5) Yijk = μ + FLSi + BMPR1Bj + TSHRk + eijk

where *Yijk* represents total litter weight from the second to the fourth parity; *μ* represents the overall mean; *FLSi* represents the effect of first-parity litter size; *BMPR1Bj* represents the effect of *BMPR1B* genotype; *TSHRk* represents the effect of *TSHR* genotype; and *eijk* represents the residual error. Model performance was summarized using *R*^2^, adjusted *R*^2^, predicted *R*^2^, root mean square error (RMSE), the overall model *F*-test, and *F*-tests for the individual fixed factors. Predicted *R*^2^ was calculated using the prediction error sum of squares (PRESS) method as 1-PRESS/SST, where SST denotes the total sum of squares. The PRESS-based predicted *R*^2^ was interpreted as an internal assessment of predictive performance rather than as independent external validation.

## 3. Results

### 3.1. Comparison of Litter Size, Litter Weight, and Lambing Type Between Hu Sheep and Tan Sheep

Across all available parity-level records, the descriptive mean litter size was 2.29 ± 0.68 in Hu sheep and 1.00 ± 0.00 in Tan sheep. Across the first four parities, the mean litter size of Tan sheep remained 1.00 ± 0.00 at each parity, with no within-population variation observed. The mean litter sizes of Hu sheep at parities 1–4 were 1.99 ± 0.59, 2.26 ± 0.52, 2.51 ± 0.68, and 2.59 ± 0.82, respectively (Table 3).

Across all available parity-level records, the descriptive mean litter weight was 8.16 ± 1.76 kg in Hu sheep and 5.17 ± 0.75 kg in Tan sheep. Analysis across different parities showed that the litter weights of Tan sheep from the first to the fourth parity were 5.01 ± 0.81, 5.03 ± 0.81, 5.43 ± 0.55, and 5.27 ± 0.73 kg, respectively. The litter weights of Hu sheep from the first to the fourth parity were 7.38 ± 1.64, 8.12 ± 1.41, 8.74 ± 1.74, and 8.79 ± 1.93 kg, respectively. A significant population-by-parity interaction was detected for litter weight (χ^2^ = 38.48, df = 3, *p* < 0.001). Parity-specific contrasts showed that litter weight was higher in the Hu sheep population at each parity, with model-estimated differences of 2.38, 3.09, 3.30, and 3.54 kg at parities 1–4, respectively (all Holm-adjusted *p* < 0.001; Table 4).

The distribution of lambing type differed between the two populations. Tan sheep had a singleton-birth proportion of 100.00% at each parity and no multiple births were recorded. In contrast, Hu sheep had multiple-birth proportions of 82.03%, 96.40%, 96.28%, and 92.31% from the first to the fourth parity, respectively. Corresponding singleton-birth proportions were 17.97%, 3.60%, 3.72%, and 7.69%, respectively (Table 5). Fisher’s exact tests showed that the distribution of singleton and multiple births differed between the two populations at each parity after Holm correction (*p* < 0.001).

### 3.2. Analysis of Changes in Litter Size and Litter Weight from the First to the Fourth Parity in Hu Sheep

**Figure 1:**
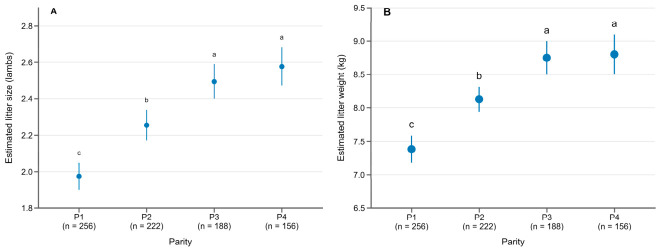
Changes in litter size and litter weight from the first to the fourth parity in Hu sheep. (**A**) Model-estimated litter size based on a zero-truncated COM–Poisson generalized linear mixed model; (**B**) model-estimated litter weight based on a heteroscedastic linear mixed-effects model. Ewe identity was included as a random effect. Points and error bars represent estimated marginal means and 95% confidence intervals, respectively. Different lowercase letters indicate significant differences among parities (*p* < 0.05).

Using all available longitudinal records, litter size and litter weight increased from the first to the third parity and remained similar between the third and fourth parities in Hu sheep. The model-estimated litter sizes at the first, second, third, and fourth parities were 1.97, 2.25, 2.49, and 2.58 lambs, respectively, whereas the corresponding model-estimated litter weights were 7.38, 8.13, 8.75, and 8.80 kg, respectively. Model-based pairwise comparisons showed that, for both traits, the first parity was significantly lower than the second, third, and fourth parities, and the second parity was significantly lower than the third and fourth parities. No significant difference was detected between the third and fourth parities (Figure 1). The complete-case analysis restricted to the 156 ewes with records for all four parities produced the same ordering of parity-specific estimates and the same pairwise significance pattern as the analysis based on all available records. Full results of the overall parity tests, estimated marginal means, and Tukey-adjusted pairwise comparisons are provided in Supplementary Table S1.

**Figure 2:**
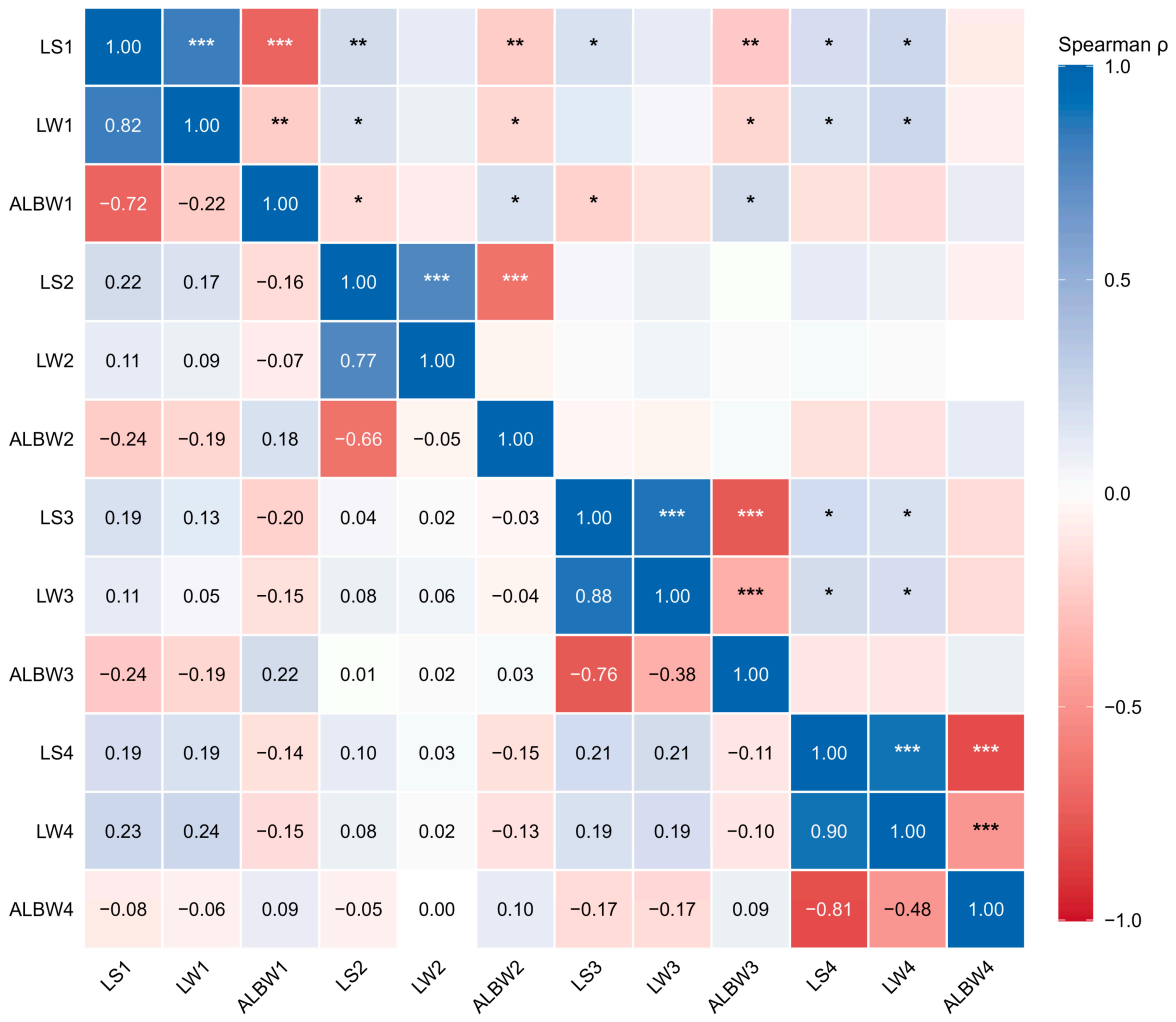
Spearman correlations among reproductive traits across the first four parities in Hu sheep. The lower triangle shows Spearman correlation coefficients (ρ), and the upper triangle shows significance after Benjamini–Hochberg false discovery rate correction. * *p* < 0.05; ** *p* < 0.01; *** *p* < 0.001. LS, litter size; LW, litter weight; ALBW, average lamb birth weight; numbers 1–4 indicate parity.

Spearman correlation analysis showed positive correlations between litter size and litter weight within each parity, with correlation coefficients of 0.82, 0.77, 0.88, and 0.90 from the first to the fourth parity, respectively. In contrast, litter size was negatively correlated with average lamb birth weight, with corresponding coefficients of −0.72, −0.66, −0.76, and −0.81. These within-parity correlations remained significant after Benjamini–Hochberg false discovery rate correction, whereas correlations between the same traits across different parities were generally smaller in magnitude (Figure 2). The complete results for all 66 pairwise correlations, including the corresponding BH-FDR-adjusted *p*-values, are provided in Supplementary Table S3. The parity-specific repeatability estimates for litter weight were 0.119, 0.156, 0.104, and 0.087 from the first to the fourth parity, respectively (Supplementary Table S4).

### 3.3. Identification and Genotyping of Candidate SNP Loci in Hu Sheep

**Figure 3:**
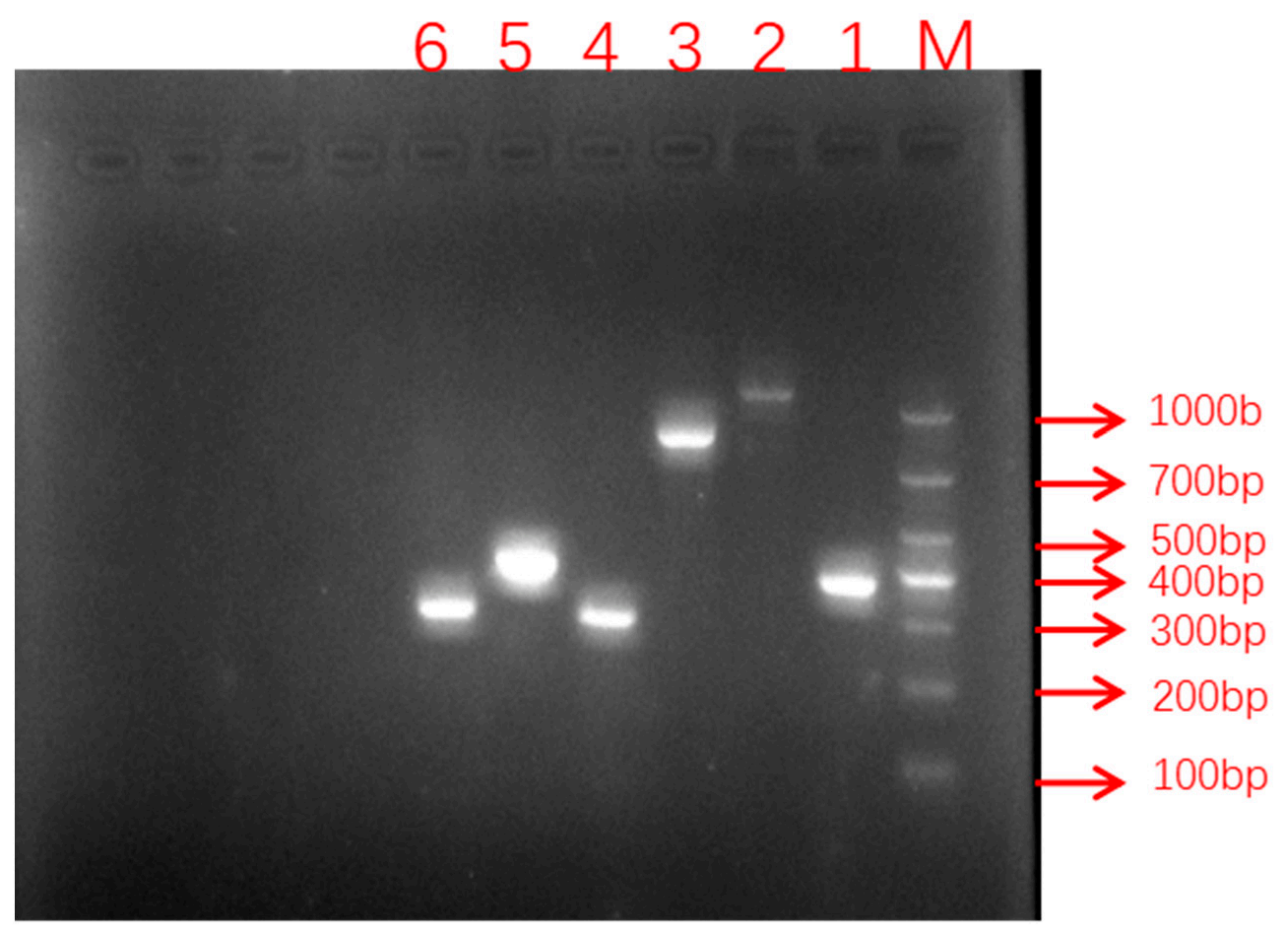
Representative PCR amplification products generated from pooled Hu sheep genomic DNA. M: DNA marker; 1: *BMPR1B* rs418841713; 2: *BMP15* rs55628000; 3: *GDF9* rs160076413; 4: *TSHR* rs406686139; 5: *GRID2* rs410806480; 6: *GRID2* rs426968202.

To screen candidate SNP loci related to litter size in Hu sheep, fragments of candidate genes including *BMPR1B*, *BMP15*, *GDF9*, *GRID2*, and *TSHR* were subjected to PCR amplification, sequencing validation, and genotyping. For preliminary evaluation of primer specificity and amplicon size, PCR amplification was performed using a pooled Hu sheep genomic DNA template. The PCR amplification results showed that a single predominant band of the expected size was obtained for each candidate fragment, supporting the specificity of the primers and their suitability for subsequent genotyping (Figure 3).

**Figure 4:**
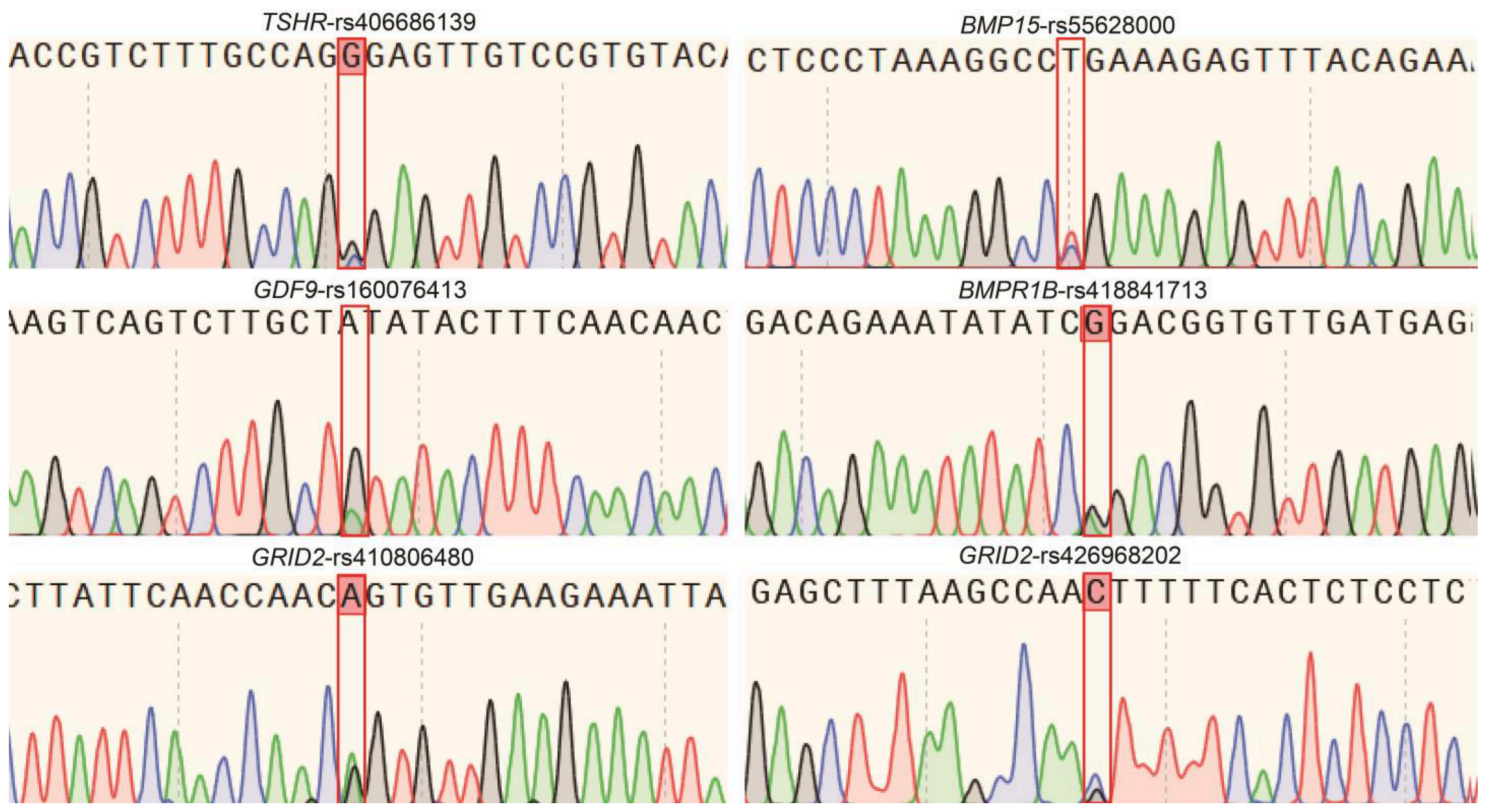
Representative Sanger sequencing chromatograms of six candidate SNP loci in Hu sheep. Red boxes indicate the target nucleotide positions. The colored chromatogram traces represent Sanger sequencing signals, and the letters above the traces indicate the corresponding nucleotide calls.

Sanger sequencing of PCR products amplified from the pooled Hu sheep DNA samples verified the target nucleotide positions of the four preselected SNPs in *BMPR1B*, *BMP15*, *GDF9*, and *TSHR* and identified the two intronic *GRID2* variants rs410806480 and rs426968202 (Figure 4). Individual-level genotypes at the six candidate SNP loci were subsequently determined using PARMS assays and used for downstream genetic diversity and association analyses.

### 3.4. Genetic Diversity Analysis of Candidate SNP Loci

Genotype and allele distributions and genetic diversity parameters for the six candidate SNP loci are summarized in Table 6. The numbers of successfully genotyped ewes ranged from 254 to 256 among the loci. Based on the PIC values, *BMPR1B* rs418841713, *BMP15* rs55628000, *GRID2* rs410806480, and *GRID2* rs426968202 showed low polymorphism, whereas *GDF9* rs160076413 and *TSHR* rs406686139 showed moderate polymorphism and had the highest PIC values among the six loci, with a PIC value of 0.375 at each locus.

*GRID2* rs426968202 showed the lowest genetic diversity and was nearly fixed for the TT genotype. Of the 255 successfully genotyped ewes, 254 carried the TT genotype and one carried the CT genotype. Exact tests detected no significant deviation from Hardy–Weinberg equilibrium at any of the six loci (*p* > 0.05). However, the HWE result for *GRID2* rs426968202 was not interpreted further because the minor allele count was 1.

### 3.5. Association Analysis Between Candidate SNP Loci and Litter Size and Litter Weight in Hu Sheep

Of the six candidate SNPs, five were included in the formal repeated-measures association analyses. *GRID2* rs426968202 was not formally tested because it was nearly fixed in the study population. No parity-by-genotype interaction was significant for either litter size or litter weight after Benjamini–Hochberg false discovery rate correction. Therefore, models without the interaction term were used to interpret the overall genotype effects.

For litter size, significant genotype associations were detected for *BMPR1B* rs418841713 and *TSHR* rs406686139. The overall genotype effect of *BMPR1B* rs418841713 was significant (LR χ^2^ = 15.672, df = 1, *p* < 0.001, BH-FDR < 0.001). Across the four parities, ewes with the GG genotype had an estimated litter size 0.257 lambs higher than that of ewes with the AG genotype (95% CI: 0.135–0.379; Tukey-adjusted *p* < 0.001).

The overall genotype effect of *TSHR* rs406686139 on litter size was also significant (LR χ^2^ = 16.828, df = 2, *p* < 0.001, BH-FDR = 0.001). Pairwise comparisons showed no significant difference between the CC and CG genotypes (*p* = 0.835). However, both CC and CG were associated with higher litter size than GG. The estimated difference was 0.210 lambs between CC and GG (95% CI: 0.040–0.379; *p* = 0.010) and 0.247 lambs between CG and GG (95% CI: 0.108–0.387; *p* < 0.001). No significant association with litter size was detected for *BMP15* rs55628000, *GDF9* rs160076413, or *GRID2* rs410806480 after BH-FDR correction (Table 7). The genotype-specific sample sizes and unadjusted descriptive litter-size means at each parity are presented in Supplementary Table S5.

For litter weight, significant genotype associations were likewise detected for *BMPR1B* rs418841713 and *TSHR* rs406686139. The overall genotype effect of *BMPR1B* rs418841713 was significant (LR χ^2^ = 10.761, df = 1, *p* = 0.001, BH-FDR = 0.003). Ewes with the GG genotype had an estimated litter weight 0.526 kg higher than that of ewes with the AG genotype (95% CI: 0.213–0.839 kg; Tukey-adjusted *p* = 0.001).

The overall genotype effect of *TSHR* rs406686139 on litter weight was also significant (LR χ^2^ = 13.564, df = 2, *p* = 0.001, BH-FDR = 0.003). No significant difference was detected between the CC and CG genotypes (*p* = 0.894). In contrast, both genotypes were associated with higher litter weight than GG. The estimated difference was 0.574 kg between CC and GG (95% CI: 0.149–1.000 kg; *p* = 0.005) and 0.502 kg between CG and GG (95% CI: 0.148–0.857 kg; *p* = 0.003). No significant association with litter weight was detected for *BMP15* rs55628000, *GDF9* rs160076413, or GRID2 rs410806480 after BH-FDR correction (Table 8). The corresponding genotype-specific sample sizes and unadjusted descriptive litter-weight means are presented in Supplementary Table S6.

The significance status and direction of the associations for *BMPR1B* rs418841713 and *TSHR* rs406686139 were unchanged in the complete-four-parity analysis, low-frequency-genotype-merging analysis, and year-adjusted analysis. Detailed comparisons between the primary and sensitivity analyses are provided in Supplementary Table S7. These findings indicate that the observed associations were robust within the present Hu sheep population, although they should not be interpreted as evidence of causality or as sufficient validation for direct marker-assisted selection.

### 3.6. Preliminary Evaluation of the Combined Genotypes of BMPR1B and TSHR in an Independent Hu Sheep Population

Based on the significant associations of the single-locus association analysis described above, this study further evaluated the combined genotypes of *BMPR1B* rs418841713 and *TSHR* rs406686139 in another independent Hu sheep population. The results showed that the distribution of different combined genotypes was uneven in the independent population. Combinations carrying the *BMPR1B* GG genotype accounted for a relatively high proportion, whereas combinations containing low-frequency genotypes had fewer individuals. Among the combined-genotype groups, GGCG had the numerically highest mean total litter size (4.11 ± 2.23), compared with GGCC (3.77 ± 1.76) and GGGG (3.83 ± 2.05). However, the differences among the evaluated combined-genotype groups did not reach statistical significance (Table 9).

Overall, the independent population analysis did not provide statistically significant evidence of differences in litter size among the evaluated *BMPR1B*–*TSHR* combined genotypes. However, the sample sizes of some combined genotypes were small. Therefore, the observed numerical differences should be interpreted as exploratory, and further confirmation in larger independent populations with more balanced genotype-group sizes is required.

### 3.7. Exploratory Evaluation of Subsequent Litter Weight Performance Using First-Parity Litter Size and Candidate Genotypes

In this study, total litter weight from the second to the fourth parity was used as the dependent variable, and a fixed-effects linear model was fitted. First-parity litter size, *BMPR1B* rs418841713 genotype, and *TSHR* rs406686139 genotype were included in the model as fixed factors. After excluding the ewe with the extremely rare *BMPR1B* AA genotype and individuals with missing *TSHR* genotypes, a total of 154 Hu sheep with complete records across all four parities and valid genotypes were used for the analysis. The overall model was statistically significant (*F* = 6.715, df = 5, 148, *p* < 0.001), with an *R*^2^ of 0.185, adjusted *R*^2^ of 0.157, predicted *R*^2^ of 0.108, and a root mean square error (RMSE) of 2.947 kg. Fixed-effect tests showed that first-parity litter size (*F* = 5.152, df = 2, *p* = 0.0069), *BMPR1B* genotype (*F* = 9.610, df = 1, *p* = 0.0023), and *TSHR* genotype (*F* = 4.462, df = 2, *p* = 0.0131) were significantly associated with total litter weight from the second to the fourth parity (Table 10).

Using ewes with one lamb at the first parity, the *BMPR1B* AG genotype, and the *TSHR* GG genotype as the reference levels, ewes with three lambs at the first parity had an estimated 2.58 kg higher total litter weight from the second to the fourth parity (*p* = 0.00195), whereas the difference between ewes with two lambs and those with one lamb at the first parity did not reach statistical significance. Individuals with the *BMPR1B* GG genotype had an estimated 1.88 kg higher total litter weight from the second to the fourth parity compared with those with the AG genotype (*p* = 0.00232). At the *TSHR* locus, individuals with the CC and CG genotypes had estimated 2.12 and 1.19 kg higher total litter weights, respectively, than those with the GG genotype (*p* = 0.00408 and *p* = 0.04254, respectively). Overall, first-parity litter size and the two candidate genotypes explained a statistically significant but limited proportion of the variation in subsequent litter weight within the present dataset. The relatively low adjusted and predicted *R*^2^ values indicate limited explanatory and internal predictive performance. Therefore, this exploratory model should not be interpreted as an independently validated tool for individual prediction or selection decisions.

## 4. Discussion

Litter size is a key economically important trait in the evaluation of reproductive performance in sheep, whereas litter weight integrates litter size and lamb birth weight and provides a complementary measure of ewe productivity within a parity [1,24]. In this study, Hu sheep had a descriptively higher litter size, significantly higher litter weight, and higher multiple-birth proportions than Tan sheep across all four parities. Hu sheep have long been noted for reproductive characteristics such as multiple lambing, early maturity, and year-round estrus, whereas Tan sheep are generally characterized by a predominantly singleton-lambing pattern. However, because the two populations originated from different farms, breed and farm effects could not be separated. Therefore, these contrasts should be interpreted as population-level phenotypic differences rather than as effects attributable solely to breed. The Tan sheep population was used as a phenotypic reference, whereas the candidate-SNP analyses were restricted to Hu sheep.

In addition to genetic background, parity is also an important factor associated with ewe litter size and litter weight [25]. In this study, the primary analysis based on all 822 available records from 256 Hu ewes showed that both litter size and litter weight increased with parity, mainly from the first to the third parity, and then tended to stabilize after the third parity. The analysis restricted to the 156 ewes with complete records across all four parities showed the same pattern. In primiparous ewes, body condition and reproductive system development may not yet be fully mature. As parity increases, ewe physiological maturity and adaptation to pregnancy may contribute to higher litter size and litter weight. Because consecutive parity records require long-term tracking of the same group of ewes, data collection is time-consuming and is easily affected by culling, missing records, and changes in management conditions. Therefore, many candidate gene studies still mainly focus on a single parity or average litter size [4,5]. Thus, multi-parity phenotypic records provide a more complete evaluation of ewe reproductive performance and reduce reliance on records from a single parity.

An increase in litter size does not necessarily result in a simultaneous increase in average lamb birth weight. In this study, Spearman correlation analysis showed that litter size in Hu sheep was positively correlated with litter weight and negatively correlated with average lamb birth weight within each parity, and these correlations remained significant after Benjamini–Hochberg false discovery rate correction. This indicates that litters containing more lambs tended to have greater total litter weight but lower average weight per lamb. This may be related to the allocation of maternal nutrient supply and uterine space among multiple fetuses [24,25]. However, because average lamb birth weight was calculated by dividing litter weight by litter size, its negative correlation with litter size may partly reflect this mathematical relationship and should not be interpreted solely as a biological trade-off. Therefore, selection for high prolificacy in Hu sheep should not focus only on litter size, but should also consider litter weight, average lamb birth weight, and performance across consecutive parities. The parity-specific repeatability estimates for litter weight were low, ranging from 0.087 to 0.156, indicating that persistent between-ewe differences accounted for only a small proportion of the total variation and further supporting the value of multi-parity records in reproductive performance evaluation.

In the candidate locus analysis, *BMPR1B* rs418841713 showed significant overall associations with both litter size and litter weight. In the repeated-measures analyses, ewes with the GG genotype had an estimated litter size 0.257 lambs higher and an estimated litter weight 0.526 kg higher than those with the AG genotype. Both associations remained significant after BH-FDR correction, and no significant parity-by-genotype interaction was detected. The direction and significance of these associations were also unchanged in the sensitivity analyses. *BMPR1B* is one of the most widely studied fecundity-related genes in sheep, and its classical FecB mutation is known to affect follicular development and ovulation rate through the BMP/SMAD signaling pathway [5,26,27,28]. The rs418841713 locus evaluated in the present study corresponds to the classical FecB variant. However, the present results provide population-level association evidence rather than direct functional validation. Therefore, although the observed association is consistent with the established biological role of FecB, independent replication is still required before its effect size and practical value in the present Hu sheep population can be determined.

*TSHR* rs406686139 also showed significant overall associations with litter size and litter weight. Ewes with the CC and CG genotypes did not differ significantly from each other, whereas both genotypes were associated with higher litter size and litter weight than the GG genotype. These associations remained significant after BH-FDR correction, and no significant parity-by-genotype interaction was detected, providing no evidence that the genotype association differed among parities. The direction and significance of the results were also consistent across the sensitivity analyses. *TSHR* may participate in the regulation of reproductive seasonality through photoperiodic responses, thyroid hormone metabolism, and the hypothalamic–pituitary–gonadal axis [19,29]. Therefore, the observed associations are biologically plausible [30]. Nevertheless, this study did not directly examine the functional effect of rs406686139, and the association may reflect either a direct effect or linkage disequilibrium with another functional variant. Accordingly, rs406686139 should be considered an associated candidate locus rather than a confirmed causal or major-effect marker.

In contrast, *BMP15* rs55628000, *GDF9* rs160076413, and *GRID2* rs410806480 showed no significant overall associations with litter size or litter weight after accounting for repeated measurements and BH-FDR correction. No significant parity-by-genotype interactions were detected for these loci. The extremely rare *BMP15* CC and *GRID2* rs410806480 AA genotypes were not retained as separate groups in the primary models, which limited the evaluation of effects specific to these rare homozygous genotypes. *BMP15* and *GDF9* are important oocyte-derived fecundity-related genes, but the effects of different mutations in these genes vary among breeds and populations [8,16,31]. Therefore, the lack of significant associations for rs55628000 and rs160076413 in this study does not exclude the biological roles of these genes in sheep reproduction. For *GRID2*, rs410806480 showed no significant association with either trait, whereas rs426968202 was nearly fixed in the current population and could not be meaningfully evaluated by formal association testing. These results do not establish the absence of a reproductive role for *GRID2*, but indicate that the two intronic variants examined here currently lack sufficient association or functional evidence to support their use as reproductive markers. Further evaluation in independent populations with adequate genotype variation, together with functional evidence, is required [32].

The results of the combined genotype analysis and fixed-effect model further indicate that the evaluated genetic and early phenotypic factors explained only part of the variation in litter size and litter weight. In the preliminary evaluation using an independent Hu sheep population, the combination of *BMPR1B* GG and *TSHR* CG showed the numerically highest mean total litter size, but the difference did not reach statistical significance. Therefore, the independent population analysis did not provide statistically significant evidence that the evaluated combined genotypes differed in litter size, and the observed numerical pattern requires confirmation in larger populations with more balanced genotype-group sizes. The exploratory fixed-effects model showed that first-parity litter size, *BMPR1B* genotype, and *TSHR* genotype were each significantly associated with total litter weight from the second to the fourth parity, but the *R*^2^ and predicted *R*^2^ of the model were low, indicating limited explanatory and internal predictive ability [33]. Thus, although these factors contributed statistically significant information within the present dataset, the model should not be interpreted as a validated tool for individual prediction or selection decisions. This result is consistent with the complex quantitative nature of reproductive traits, which are influenced by multiple genetic and non-genetic factors [24,25].

Several limitations should be considered when interpreting the present findings. First, the Hu and Tan sheep populations were obtained from different farms, and population and farm effects could therefore not be separated. Second, detailed individual-level information on nutrition, body condition, management exposure, and pedigree relationships was not available in the analysis dataset, and residual environmental and genetic confounding cannot be excluded. Third, the candidate-gene design evaluated only a limited number of loci, while extremely rare or nearly fixed genotypes reduced the precision and statistical power of some comparisons. Except for the established FecB variant, the observed SNP associations were not supported by direct functional validation and should not be interpreted as evidence of causality. Finally, the separate Hu sheep population contained incomplete later-parity records and unbalanced combined-genotype group sizes, and the fixed-effect model was evaluated internally rather than in an external validation population. Therefore, the combined-genotype and prediction findings should be regarded as exploratory and require replication in larger independent populations with more complete phenotypic, environmental, pedigree, and genome-wide marker information.

In summary, the Hu and Tan sheep populations showed marked phenotypic differences in litter size, litter weight, and lambing type. In Hu sheep, both traits increased from the first to the third parity and then tended to stabilize, highlighting the value of consecutive parity records. *BMPR1B* rs418841713 and *TSHR* rs406686139 were significantly associated with both litter size and litter weight, with higher values observed for the *BMPR1B* GG and *TSHR* CC/CG genotypes. The significance status and effect directions of these associations were unchanged across the complete-case, rare-genotype-coding, and year-adjusted sensitivity analyses. Although the *BMPR1B*–*TSHR* combined-genotype groups did not differ significantly in another Hu sheep population, GGCG showed the numerically highest mean total litter size. The exploratory model also indicated that first-parity litter size and the two genotypes contributed information on subsequent litter weight, although its individual-level predictive performance was limited. Overall, integrating multi-parity records with candidate-genotype information provides a basis for further validation and more comprehensive reproductive evaluation in Hu sheep.

## 5. Conclusions

In conclusion, litter size and litter weight in Hu sheep increased from the first to the third parity and then tended to stabilize, supporting the value of consecutive parity records for reproductive-performance evaluation. *BMPR1B* rs418841713 and *TSHR* rs406686139 were significantly associated with both litter size and litter weight in the repeated-measures analyses, and the significance status and effect directions of these associations were unchanged across the complete-case, rare-genotype-coding, and year-adjusted sensitivity analyses. However, the *BMPR1B*–*TSHR* combined-genotype groups did not differ significantly in another Hu sheep population, and the exploratory fixed-effect model showed limited internal predictive performance. Therefore, multi-parity phenotypic records and these candidate loci provide a basis for further evaluation, but the combined-genotype and prediction findings require independent replication before being considered for individual selection decisions.

## Figures and Tables

**Table 1:** Primer sequences used for PCR amplification of candidate gene fragments.

Primer Name	Primer Sequences (5′→3′)	Annealing Temperature Tm (°C)	Product Size (bp)
*BMPR1B*	F: CGCACTAACAGTGTGTTGGG	56	378
	R: AGCAAATAACATTCTTGGGCTCAT		
*GDF9*	F: TTAATTCACCACCAAAGCTATT	55	878
	R: GGGAAAAAGAAACTGTTTGAAG		
*BMP15*	F: ATTCATTTCAGGGCTGCTTG	55	1104
	R: CAATGCTGAAGGCAAGGAAT		
*TSHR*	F: GTATCTGCTCCTCATCGCCT	55	293
	R: AGCTGCTTATTCCCACCAAAG		
*GRID2*	F: GAGATTTTGGTGTGCTGGTGT	57	410
	R: TCCAAGGCCTTTACTCAGTGA		
*GRID2*	F: GGAGGTCAGGAAGTAAAGGGT	57	305
	R: TGAAGTGATGGGACTGGGTG		

**Table 2:** Primer sequences used for SNP genotyping.

SNP	Primers	Primer Sequence	Allele
rs55628000	*BMP15-Ra*	GAAGGTGACCAAGTTCATGCTGGGTCTTTTTCTGTAAACTCTTTCA	T
	*BMP15-Rg*	GAAGGTCGGAGTCAACGGATTGGGTCTTTTTCTGTAAACTCTTTCG	C
	*BMP15-F*	AGTGTTCAGAAGACCAAACCTCTC	
rs418841713	*BMPR1B-Rt*	GAAGGTGACCAAGTTCATGCTCATGCCTCATCAACACCGTCT	A
	*BMPR1B-Rc*	GAAGGTCGGAGTCAACGGATTATGCCTCATCAACACCGTCC	G
	*BMPR1B-F*	AAGTGTTCTTCACTACAGAGGAGGC	
rs160076413	*GDF9-Rt*	GAAGGTGACCAAGTTCATGCTAAAAGAAATGGAGTTGTTGAAAGTATAT	A
	*GDF9-Rc*	GAAGGTCGGAGTCAACGGATTAAAAGAAATGGAGTTGTTGAAAGTATAC	G
	*GDF9-F*	ACTGTTGTGGAACATTTATTCAAGTC	
rs410806480	*GRID2-480Fg*	GAAGGTGACCAAGTTCATGCTCAAGAAGCTTTTTAGTGAAGATTAAATG	G
	*GRID2-480Fa*	GAAGGTCGGAGTCAACGGATTTCAAGAAGCTTTTTAGTGAAGATTAAATA	A
	*GRID2-480R*	AAGCCTCTATTTGTTGAGTATTTACTAATG	
rs426968202	*GRID2-202Ra*	GAAGGTGACCAAGTTCATGCTTCAGAATTCCACATGAAGTTTAACA	T
	*GRID2-202Rg*	GAAGGTCGGAGTCAACGGATTCAGAATTCCACATGAAGTTTAACG	C
	*GRID2-202F*	GAGTCTAGAAAACATACCTGAATAGCTC	
rs406686139	*TSHR-Fc*	GAAGGTGACCAAGTTCATGCTTCTTCACCGTCTTTGCCAGC	C
	*TSHR-Fg*	GAAGGTCGGAGTCAACGGATTTCTTCACCGTCTTTGCCAGG	G
	*TSHR-R*	GGATCTTGCGGTCCAGGTG	

**Table 3:** Comparison of litter size between Hu sheep and Tan sheep at different parities.

Breed	Overall Mean Litter Size	Parity 1	Parity 2	Parity 3	Parity 4
Tan sheep	1.00 ± 0.00	1.00 ± 0.00	1.00 ± 0.00	1.00 ± 0.00	1.00 ± 0.00
Hu sheep	2.29 ± 0.68	1.99 ± 0.59	2.26 ± 0.52	2.51 ± 0.68	2.59 ± 0.82

Note: Values are presented as mean ± standard deviation. The numbers of parity-level records at parities 1–4 were 256, 222, 188, and 156 for Hu sheep and 90, 87, 83, and 58 for Tan sheep, respectively. Overall means were calculated using all available parity-level records.

**Table 4:** Comparison of litter weight between Hu sheep and Tan sheep at different parities.

Breed	Overall Mean Litter Weight (kg)	Parity 1	Parity 2	Parity 3	Parity 4
Tan sheep	5.17 ± 0.75	5.01 ± 0.81 ^B^	5.03 ± 0.81 ^B^	5.43 ± 0.55 ^B^	5.27 ± 0.73 ^B^
Hu sheep	8.16 ± 1.76	7.38 ± 1.64 ^A^	8.12 ± 1.41 ^A^	8.74 ± 1.74 ^A^	8.79 ± 1.93 ^A^

Note: Values are presented as mean ± standard deviation. Within each parity, different uppercase superscript letters indicate significant differences between the two populations based on contrasts derived from a heteroscedastic linear mixed-effects model. *p* values for the four parity-specific comparisons were adjusted using the Holm method. Overall means were calculated using all available parity-level records.

**Table 5:** Distribution of lambing type in Hu sheep and Tan sheep at different parities.

Breed	Parity	Total (No.)	Singleton Birth (No., %)	Multiple Birth (No., %)	Holm-Adjusted *p*
Tan sheep	1	90	90 (100.00%)	0 (0.00%)	<0.001
	2	87	87 (100.00%)	0 (0.00%)	<0.001
	3	83	83 (100.00%)	0 (0.00%)	<0.001
	4	58	58 (100.00%)	0 (0.00%)	<0.001
Hu sheep	1	256	46 (17.97%)	210 (82.03%)	
	2	222	8 (3.60%)	214 (96.40%)	
	3	188	7 (3.72%)	181 (96.28%)	
	4	156	12 (7.69%)	144 (92.31%)	

Note: Singleton birth was defined as a litter size of 1, whereas multiple birth was defined as a litter size of ≥2. Differences between the two populations within each parity were evaluated using Fisher’s exact tests, and the resulting *p* values were adjusted using the Holm method.

**Table 6:** Genetic diversity analysis of candidate SNP loci in Hu sheep.

Locus	Successfully Genotyped, *n*	Missing, *n*	Genotype Frequency	Allele Frequency	He	Ho	Ne	PIC	P_HWE_
*BMPR1B* rs418841713	256	0	AA: 0.004 /AG: 0.195 /GG: 0.801	A: 0.102/G: 0.898	0.182	0.195	1.223	0.166	0.488
*BMP15* rs55628000	254	2	CC: 0.012/CT: 0.252/TT: 0.736	C: 0.138/T: 0.862	0.238	0.252	1.312	0.209	0.436
*GDF9* rs160076413	255	1	AA: 0.255/AG: 0.467/GG: 0.278	A: 0.488/G: 0.512	0.500	0.467	1.999	0.375	0.316
*GRID2* rs410806480	255	1	AA: 0.004 /AG: 0.161 /GG: 0.835	A: 0.084/G: 0.916	0.154	0.161	1.183	0.142	1.000
*GRID2* rs426968202	255	1	CC: 0.000 /CT: 0.004 /TT: 0.996	C: 0.002/T: 0.998	0.004	0.004	1.004	0.004	1.000
*TSHR* rs406686139	254	2	CC: 0.232/CG: 0.508/GG: 0.260	C: 0.486/G: 0.514	0.500	0.508	1.998	0.375	0.900

Note: Genotype frequencies were calculated using the number of successfully genotyped ewes at each locus as the denominator. He, expected heterozygosity; Ho, observed heterozygosity; Ne, effective number of alleles; PIC, polymorphism information content; P_HWE_, *p* value from the exact test for Hardy–Weinberg equilibrium. The HWE test for *GRID2* rs426968202 had limited informativeness because the minor allele count was 1.

**Table 7:** Association analysis between candidate SNP loci and litter size in Hu sheep.

Gene	SNP ID	Genotype Groups	Ewes, *n*	Records, *n*	Genotype Effect, LR χ^2^ (df)	*p*-Value	BH-FDR	Parity × Genotype, LR χ^2^ (df)	Interaction *p*-Value	Interaction BH-FDR	Result
*BMPR1B*	rs418841713	AG and GG †	255	818	15.672 (1)	<0.001	<0.001	3.102 (3)	0.376	0.761	Significant; GG > AG
*BMP15*	rs55628000	CT and TT †	251	806	0.0004 (1)	0.984	0.984	2.346 (3)	0.504	0.761	Not significant
*GDF9*	rs160076413	AA, AG, and GG	255	818	2.088 (2)	0.352	0.464	4.717 (6)	0.581	0.761	Not significant
*GRID2*	rs410806480	AG and GG †	254	816	0.799 (1)	0.371	0.464	3.814 (3)	0.282	0.761	Not significant
*GRID2*	rs426968202	TT and CT ‡	255	818	-	-	-	-	-	-	Not formally tested ‡
*TSHR*	rs406686139	CC, CG, and GG	254	815	16.828 (2)	<0.001	0.001	4.503 (6)	0.609	0.761	Significant; CC ≈ CG > GG

Note: *p*-values represent the overall genotype effects and parity-by-genotype interaction effects obtained from likelihood-ratio tests; BH-FDR values are Benjamini–Hochberg-adjusted *p*-values. Genotype-effect directions were determined from Tukey-adjusted pairwise comparisons. † The rare *BMPR1B* AA, *BMP15* CC, and *GRID2* rs410806480 AA genotypes were excluded as separate groups from the primary models. ‡ *GRID2* rs426968202 was nearly fixed and was not formally tested. LR, likelihood ratio; BH-FDR, Benjamini–Hochberg false discovery rate.

**Table 8:** Association analysis between candidate SNP loci and litter weight in Hu sheep.

Gene	SNP ID	Genotype Groups	Ewes, *n*	Records, *n*	Genotype Effect, LR χ^2^ (df)	*p*-Value	BH-FDR	Parity × Genotype, LR χ^2^ (df)	Interaction *p*-Value	Interaction BH-FDR	Result
*BMPR1B*	rs418841713	AG and GG †	255	818	10.761 (1)	0.001	0.003	2.499 (3)	0.475	0.761	Significant; GG > AG
*BMP15*	rs55628000	CT and TT †	251	806	0.092 (1)	0.762	0.846	0.992 (3)	0.803	0.882	Not significant
*GDF9*	rs160076413	AA, AG, and GG	255	818	2.127 (2)	0.345	0.464	6.556 (6)	0.364	0.761	Not significant
*GRID2*	rs410806480	AG and GG †	254	816	1.813 (1)	0.178	0.356	2.970 (3)	0.396	0.761	Not significant
*GRID2*	rs426968202	TT and CT ‡	255	818	-	-	-	-	-	-	Not formally tested ‡
*TSHR*	rs406686139	CC, CG, and GG	254	815	13.564 (2)	0.001	0.003	2.378 (6)	0.882	0.882	Significant; CC ≈ CG > GG

Note: *p*-values represent the overall genotype effects and parity-by-genotype interaction effects obtained from likelihood-ratio tests; BH-FDR values are Benjamini–Hochberg-adjusted *p*-values. Genotype-effect directions were determined from Tukey-adjusted pairwise comparisons. † The rare *BMPR1B* AA, *BMP15* CC, and *GRID2* rs410806480 AA genotypes were excluded as separate groups from the primary models. ‡ *GRID2* rs426968202 was nearly fixed and was not formally tested. LR, likelihood ratio; BH-FDR, Benjamini–Hochberg false discovery rate.

**Table 9:** Relationship between combined genotypes of *BMPR1B* and *TSHR* and litter size in an independent Hu sheep population.

Type	Genotype	Parity 1 *n*	Parity 1 Mean ± SD	Parity 2 *n*	Parity 2 Mean ± SD	Parity 3 *n*	Parity 3 Mean ± SD	Total Litter Size *n*	Total Litter Size Mean ± SD	*p*-Value
Single-locus favorable genotype	*BMPR1B*-GG	188	2.46 ± 0.71	88	2.50 ± 0.71	24	2.58 ± 0.97	188	3.96 ± 2.09	ns
Single-locus favorable genotype	*TSHR*-CG	99	2.46 ± 0.69	48	2.50 ± 0.77	12	2.58 ± 0.90	99	3.99 ± 2.20	ns
Combined genotype	GGCC	31	2.55 ± 0.62	12	2.67 ± 0.78	3	2.00 ± 1.00	31	3.77 ± 1.76	ns
Combined genotype	GGCG	91	2.52 ± 0.69	45	2.53 ± 0.79	12	2.58 ± 0.90	91	4.11 ± 2.23	ns
Combined genotype	GGGG	66	2.33 ± 0.77	31	2.39 ± 0.56	9	2.78 ± 1.09	66	3.83 ± 2.05	ns

Note: ns, not significant (*p* ≥ 0.05).

**Table 10:** Fixed-effects linear model evaluating the associations of first-parity litter size and candidate genotypes with total litter weight from the second to the fourth parity in Hu sheep.

Source	df	*F* Value	*p* Value	*R* ^2^	Adjusted *R*^2^	Predicted *R*^2^	RMSE (kg)
Overall model	5, 148	6.715	<0.001	0.185	0.157	0.108	2.947
First-parity litter size	2	5.152	0.0069	—	—	—	—
*BMPR1B* genotype	1	9.610	0.0023	—	—	—	—
*TSHR* genotype	2	4.462	0.0131	—	—	—	—

Note: A total of 154 Hu sheep with complete parity records and valid *BMPR1B* and *TSHR* genotypes were included in this model. RMSE indicates root mean square error; Adjusted *R*^2^ indicates adjusted coefficient of determination; Predicted *R*^2^ indicates the PRESS-based predicted coefficient of determination. For the overall model, “5, 148” represents the numerator and denominator degrees of freedom, respectively.

## Data Availability

The original contributions presented in this study are included in the article/Supplementary Materials. Further inquiries can be directed to the corresponding author.

## Supplementary Materials

The following supporting information can be downloaded at https://www.mdpi.com/article/10.3390/ani16152365/s1: Supplementary Methods: Principal R functions, model specifications, and multiple-testing procedures used in the final analyses; Table S1: Overall tests, estimated marginal means, and pairwise comparisons for parity effects on litter size and litter weight in Hu sheep; Table S2: Model selection, diagnostic assessment, and sensitivity-analysis results for the litter-size and litter-weight models; Table S3: Pairwise Spearman correlations among 12 parity-specific reproductive traits in Hu sheep; Table S4: Variance components and parity-specific repeatability estimates for litter weight in Hu sheep; Table S5: Genotype-specific sample sizes and descriptive litter-size statistics across parities in Hu sheep; Table S6: Genotype-specific sample sizes and descriptive litter-weight statistics across parities in Hu sheep; Table S7: Comparison of the primary and sensitivity analyses of candidate-SNP associations with litter size and litter weight in Hu sheep.
