# Supplementary material for: Screening of Candidate SNPs Potentially Associated with Multi-Parity Litter Size and Litter Weight in Hu Sheep
Source: Animals (Basel). 2026 Aug 2;16(15):2365. doi: 10.3390/ani16152365 (PMC13465654; doi:10.3390/ani16152365)
Supplement: Supplementary file 1 [file animals-16-02365-s001.zip › animals-4424412-Supplementary Methods.pdf]

```
#Supplementary Methods
#Data preparation and factor coding
dat <- analysis822 %>%
  transmute(
    Animal_ID = factor(as.character(Animal_ID)),
    Parity = factor(
      as.character(Parity),
      levels = c("P1", "P2", "P3", "P4"),
      ordered = FALSE
    ),
    Parity_number = as.integer(Parity_number),
    LS = as.integer(LS),
    LW = as.numeric(LW)
  )
genotype_levels <- list(
  BMPR1B_rs418841713 = c("AG", "GG"),
  BMP15_rs55628000 = c("CT", "TT"),
  GDF9_rs160076413 = c("AA", "AG", "GG"),
  GRID2_rs410806480 = c("AG", "GG"),
  TSHR_rs406686139 = c("CC", "CG", "GG")
)
model_data <- analysis822 %>%
  filter(!is.na(.data[[current_group_column]])) %>%
  transmute(
    Animal_ID = factor(as.character(Animal_ID)),
    Parity = factor(as.character(Parity), levels = paste0("P", 1:4)),
    SNP_Genotype = factor(
      as.character(.data[[current_group_column]]),
      levels = genotype_levels[[current_snp]]
    ),
    LS = as.integer(LS),
    LW = as.numeric(LW)
   )
# Parity effects on litter size
ls_full <- glmmTMB::glmmTMB(
  LS ~ Parity + (1 | Animal_ID),
  family = glmmTMB::truncated_compois(link = "log"),
  data = dat
)
ls_null <- glmmTMB::glmmTMB(
  LS ~ 1 + (1 | Animal_ID),
  family = glmmTMB::truncated_compois(link = "log"),
  data = dat
)
ls_lrt <- stats::anova(ls_null, ls_full)
ls_emm <- emmeans::emmeans(
  ls_full,
  ~ Parity,
  type = "response"
)
ls_pairs <- emmeans::contrast(
  ls_emm,
  method = "pairwise",
```

```
  adjust = "tukey",
  type = "response"
)
# Run simulation-based diagnostics
set.seed(20260722L)
ls_residuals <- DHARMa::simulateResiduals(
  fittedModel = ls_full,
  n = 5000L,
  refit = FALSE,
  seed = 20260722L
)
DHARMa::testUniformity(ls_residuals)
DHARMa::testDispersion(ls_residuals)
DHARMa::testOutliers(ls_residuals)

#Parity effects on litter weight
lrt <- function(reduced_model, full_model) {
  reduced_loglik <- logLik(reduced_model)
  full_loglik <- logLik(full_model)
  statistic <- 2 * (as.numeric(full_loglik) - as.numeric(reduced_loglik))
  df <- attr(full_loglik, "df") - attr(reduced_loglik, "df")
  data.frame(
    Chi_square = statistic,
    df = df,
    P_value = pchisq(statistic, df = df, lower.tail = FALSE)
  )
}
lw_homoscedastic_ml <- nlme::lme(
  fixed = LW ~ Parity,
  random = ~ 1 | Animal_ID,
  data = dat,
  method = "ML",
  na.action = na.omit
)
lw_heteroscedastic_ml <- nlme::lme(
  fixed = LW ~ Parity,
  random = ~ 1 | Animal_ID,
  weights = nlme::varIdent(form = ~ 1 | Parity),
  data = dat,
  method = "ML",
  na.action = na.omit
)
lw_null_ml <- nlme::lme(
  fixed = LW ~ 1,
  random = ~ 1 | Animal_ID,
  weights = nlme::varIdent(form = ~ 1 | Parity),
  data = dat,
  method = "ML",
  na.action = na.omit
)
variance_structure_test <- lrt(lw_homoscedastic_ml, lw_heteroscedastic_ml)
parity_test <- lrt(lw_null_ml, lw_heteroscedastic_ml)
# Estimate effects under REML
```

```
lw_final <- nlme::lme(
  fixed = LW ~ Parity,
  random = ~ 1 | Animal_ID,
  weights = nlme::varIdent(form = ~ 1 | Parity),
  data = dat,
  method = "REML",
  na.action = na.omit
)
lw_emm <- emmeans::emmeans(lw_final, ~ Parity)
lw_pairs <- emmeans::contrast(
  lw_emm,
  method = "pairwise",
  adjust = "tukey"
)
lw_residuals <- residuals(lw_final, type = "normalized")
stats::shapiro.test(lw_residuals)
stats::fligner.test(lw_residuals ~ nlme::getData(lw_final)$Parity)

#Complete four-parity sensitivity analysis
complete_id_check <- analysis822 %>%
  group_by(Animal_ID) %>%
  summarise(
    N_records = n(),
    N_distinct_parities = n_distinct(Parity),
    Has_P1 = any(Parity == "P1"),
    Has_P2 = any(Parity == "P2"),
    Has_P3 = any(Parity == "P3"),
    Has_P4 = any(Parity == "P4"),
    Complete_P1_P4 = N_records == 4 & N_distinct_parities == 4 &
      Has_P1 & Has_P2 & Has_P3 & Has_P4,
    .groups = "drop"
  )
complete_ids <- complete_id_check %>%
  filter(Complete_P1_P4) %>%
  pull(Animal_ID)
complete_data <- analysis822 %>%
  filter(Animal_ID %in% complete_ids) %>%
  arrange(Animal_ID, Parity_number)
stopifnot(
  length(complete_ids) == 156,
  nrow(complete_data) == 624,
  all(table(complete_data$Parity) == 156L)
)
ls_complete <- update(ls_full, data = complete_data)
ls_complete_null <- update(ls_null, data = complete_data)
lw_complete <- update(lw_final, data = complete_data)

#Litter-weight repeatability
extract_lw_repeatability <- function(model, analysis_label) {
  ewe_variance <- as.numeric(
    nlme::getVarCov(model, type = "random.effects")
  )[1]
  variance_weights <- nlme::varWeights(
```

```
    model$modelStruct$varStruct
  )
  model_data <- model$data
  residual_by_parity <- data.frame(
    Parity = as.character(model_data$Parity),
    Residual_variance = model$sigma^2 / variance_weights^2
  ) %>%
    group_by(Parity) %>%
    summarise(
      Residual_variance = mean(Residual_variance),
      .groups = "drop"
    ) %>%
    mutate(
      Parity = factor(Parity, levels = paste0("P", 1:4))
    ) %>%
    arrange(Parity)
  residual_by_parity %>%
    mutate(
      Analysis_population = analysis_label,
      Ewe_variance = ewe_variance,
      Repeatability = Ewe_variance /
        (Ewe_variance + Residual_variance)
    )
}
lw_repeatability_all <- extract_lw_repeatability(
  lw_final,
  "All available records"
)
lw_repeatability_complete <- extract_lw_repeatability(
  lw_complete,
  "Complete P1-P4 records"
)

#Genetic diversity and Hardy–Weinberg equilibrium
hwe_exact_wigginton <- function(obs_hom1, obs_hets, obs_hom2) {
  counts <- as.integer(c(obs_hom1, obs_hets, obs_hom2))
  if (any(is.na(counts))) stop("HWE genotype counts contain NA.")
  if (any(counts < 0)) stop("HWE genotype counts cannot be negative.")
  n_genotypes <- sum(counts)
  if (n_genotypes == 0) return(NA_real_)
  obs_homr <- min(obs_hom1, obs_hom2)
  obs_homc <- max(obs_hom1, obs_hom2)
  rare_allele_copies <- 2L * obs_homr + obs_hets
  het_probabilities <- numeric(rare_allele_copies + 1L)
  midpoint <- floor(
    rare_allele_copies *
      (2L * n_genotypes - rare_allele_copies) /
      (2L * n_genotypes)
  )
  if ((rare_allele_copies - midpoint) %% 2L != 0L) {
    midpoint <- midpoint + 1L
  }
  current_hets <- midpoint
```

```
  current_homr <- (rare_allele_copies - midpoint) / 2
  current_homc <- n_genotypes - current_hets - current_homr
  het_probabilities[current_hets + 1L] <- 1
  probability_sum <- 1
  while (current_hets > 1L) {
    next_hets <- current_hets - 2L
    het_probabilities[next_hets + 1L] <-
      het_probabilities[current_hets + 1L] *
      current_hets * (current_hets - 1L) /
      (4 * (current_homr + 1) * (current_homc + 1))
    probability_sum <- probability_sum +
      het_probabilities[next_hets + 1L]
    current_hets <- next_hets
    current_homr <- current_homr + 1
    current_homc <- current_homc + 1
  }
  current_hets <- midpoint
  current_homr <- (rare_allele_copies - midpoint) / 2
  current_homc <- n_genotypes - current_hets - current_homr
  while (current_hets <= rare_allele_copies - 2L) {
    next_hets <- current_hets + 2L
    het_probabilities[next_hets + 1L] <-
      het_probabilities[current_hets + 1L] *
      4 * current_homr * current_homc /
      (next_hets * (next_hets - 1L))
    probability_sum <- probability_sum +
      het_probabilities[next_hets + 1L]
    current_hets <- next_hets
    current_homr <- current_homr - 1
    current_homc <- current_homc - 1
  }
  het_probabilities <- het_probabilities / probability_sum
  observed_probability <- het_probabilities[obs_hets + 1L]
  exact_p <- sum(
    het_probabilities[
      het_probabilities <= observed_probability + 1e-12
    ]
  )
  min(1, exact_p)
}
observed_counts <- c(
  sum(valid_genotype == hom1),
  sum(valid_genotype == het),
  sum(valid_genotype == hom2)
)
n_valid <- length(valid_genotype)
allele1_count <- 2 * observed_counts[1] + observed_counts[2]
allele2_count <- 2 * observed_counts[3] + observed_counts[2]
p <- allele1_count / (2 * n_valid)
q <- allele2_count / (2 * n_valid)
Ho <- observed_counts[2] / n_valid
He <- 1 - (p^2 + q^2)
Ne <- 1 / (p^2 + q^2)
```

```
PIC <- 1 - p^2 - q^2 - 2 * p^2 * q^2
HWE_exact_P <- hwe_exact_wigginton(
  observed_counts[1],
  observed_counts[2],
  observed_counts[3]
)

#Primary candidate-SNP association models
ls_null <- glmmTMB::glmmTMB(
  LS ~ Parity + (1 | Animal_ID),
  family = glmmTMB::truncated_compois(link = "log"),
  data = model_data,
  control = glmm_control
)
ls_main <- glmmTMB::glmmTMB(
  LS ~ Parity + SNP_Genotype + (1 | Animal_ID),
  family = glmmTMB::truncated_compois(link = "log"),
  data = model_data,
  control = glmm_control
)
ls_interaction <- glmmTMB::glmmTMB(
  LS ~ Parity * SNP_Genotype + (1 | Animal_ID),
  family = glmmTMB::truncated_compois(link = "log"),
  data = model_data,
  control = glmm_control
)
ls_main_lrt <- anova(ls_null, ls_main, test = "Chisq")
ls_interaction_lrt <- anova(ls_main, ls_interaction, test = "Chisq")
ls_grid <- emmeans::ref_grid(
  ls_main,
  data = model_data,
  component = "cmean"
)
ls_genotype_emm <- emmeans::emmeans(
  ls_grid,
  ~ SNP_Genotype,
  weights = "equal"
)
ls_genotype_pairs <- pairs(
  ls_genotype_emm,
  adjust = "tukey"
)
lw_null_ml <- nlme::lme(
  fixed = LW ~ Parity,
  random = ~ 1 | Animal_ID,
  weights = nlme::varIdent(form = ~ 1 | Parity),
  data = model_data,
  method = "ML"
)
lw_main_ml <- nlme::lme(
  fixed = LW ~ Parity + SNP_Genotype,
  random = ~ 1 | Animal_ID,
  weights = nlme::varIdent(form = ~ 1 | Parity),
```

```
  data = model_data,
  method = "ML"
)
lw_interaction_ml <- nlme::lme(
  fixed = LW ~ Parity * SNP_Genotype,
  random = ~ 1 | Animal_ID,
  weights = nlme::varIdent(form = ~ 1 | Parity),
  data = model_data,
  method = "ML"
)
lw_main_lrt <- anova(lw_null_ml, lw_main_ml)
lw_interaction_lrt <- anova(lw_main_ml, lw_interaction_ml)
lw_main_reml <- nlme::lme(
  fixed = LW ~ Parity + SNP_Genotype,
  random = ~ 1 | Animal_ID,
  weights = nlme::varIdent(form = ~ 1 | Parity),
  data = model_data,
  method = "REML"
)
lw_interaction_reml <- nlme::lme(
  fixed = LW ~ Parity * SNP_Genotype,
  random = ~ 1 | Animal_ID,
  weights = nlme::varIdent(form = ~ 1 | Parity),
  data = model_data,
  method = "REML"
)
lw_genotype_emm <- emmeans::emmeans(
  lw_main_reml,
  ~ SNP_Genotype,
  weights = "equal"
)
lw_genotype_pairs <- pairs(
  lw_genotype_emm,
  adjust = "tukey"
)
lw_by_parity <- emmeans::emmeans(
  lw_interaction_reml,
  ~ SNP_Genotype | Parity
)
lw_within_parity <- summary(
  pairs(lw_by_parity, by = "Parity", adjust = "none"),
  by = NULL,
  adjust = "holm"
)

#Rare-genotype handling
primary_levels <- list(
  BMPR1B_rs418841713 = c("AG", "GG"),
  BMP15_rs55628000 = c("CT", "TT"),
  GRID2_rs410806480 = c("AG", "GG"),
  GDF9_rs160076413 = c("AA", "AG", "GG"),
  TSHR_rs406686139 = c("CC", "CG", "GG")
)
```

```
# Rare-genotype merge sensitivity rules
merge_spec <- list(
  BMPR1B_rs418841713 = list(
    merged_genotypes = c("AA", "AG"),
    merged_label = "AA_or_AG",
    comparison_genotype = "GG"
  ),
  BMP15_rs55628000 = list(
    merged_genotypes = c("CC", "CT"),
    merged_label = "CC_or_CT",
    comparison_genotype = "TT"
  ),
  GRID2_rs410806480 = list(
    merged_genotypes = c("AA", "AG"),
    merged_label = "AA_or_AG",
    comparison_genotype = "GG"
  )
)
grouped_values <- ifelse(
  raw_values %in% current_spec$merged_genotypes,
  current_spec$merged_label,
  ifelse(
    raw_values == current_spec$comparison_genotype,
    current_spec$comparison_genotype,
    NA_character_
  )
)

#Multiple-testing correction
global_tests <- global_tests %>%
  group_by(Test_type) %>%
  mutate(
    BH_FDR = p.adjust(
      Raw_P,
      method = "BH"
    )
  ) %>%
   ungroup()

#SNP sensitivity analyses
complete_model_data <- complete_data %>%
  filter(!is.na(.data[[current_group_column]])) %>%
  mutate(
    Animal_ID = factor(Animal_ID),
    Parity = factor(Parity, levels = paste0("P", 1:4)),
    SNP_Genotype = factor(
      .data[[current_group_column]],
      levels = genotype_levels[[current_snp]]
    )
  )
year_data <- analysis822 %>%
  filter(!is.na(Lambing_Year_numeric)) %>%
```

```
    mutate(
      Lambing_Year = factor(
        Lambing_Year_numeric,
        levels = sort(unique(Lambing_Year_numeric))
      )
    )
  year_model_data <- year_data %>%
    filter(!is.na(.data[[current_group_column]])) %>%
    mutate(
      SNP_Genotype = factor(
        .data[[current_group_column]],
        levels = genotype_levels[[current_snp]]
      )
    )
  ls_year_main <- glmmTMB::glmmTMB(
    LS ~ Parity + Lambing_Year + SNP_Genotype + (1 | Animal_ID),
    family = glmmTMB::truncated_compois(link = "log"),
    data = year_model_data,
    control = glmm_control
  )
  ls_year_interaction <- glmmTMB::glmmTMB(
    LS ~ Parity * SNP_Genotype + Lambing_Year + (1 | Animal_ID),
    family = glmmTMB::truncated_compois(link = "log"),
    data = year_model_data,
    control = glmm_control
  )
  lw_year_main_ml <- nlme::lme(
    fixed = LW ~ Parity + Lambing_Year + SNP_Genotype,
    random = ~ 1 | Animal_ID,
    weights = nlme::varIdent(form = ~ 1 | Parity),
    data = year_model_data,
    method = "ML"
  )
  lw_year_interaction_ml <- nlme::lme(
    fixed = LW ~ Parity * SNP_Genotype + Lambing_Year,
    random = ~ 1 | Animal_ID,
    weights = nlme::varIdent(form = ~ 1 | Parity),
    data = year_model_data,
    method = "ML"
  )
```
